# Reporting of sexual and gender-based violence and associated factors among survivors in Mayuge, Uganda

**DOI:** 10.4314/ahs.v22i1.8

**Published:** 2022-03

**Authors:** Jacquellyn Nambi Ssanyu, Noel Namuhani, Christine Kayemba Nalwadda

**Affiliations:** Makerere University, School of Public Health P.O. Box 22864, Kampala Uganda. Tel: +256 414 269 003

**Keywords:** Sexual and Gender-Based Violence, survivors, Mayuge, Uganda

## Abstract

**Background:**

Reporting of Sexual and Gender-Based Violence (SGBV) allows survivors to access support services to minimize the impact of the violence on their lives. However, research shows that most SGBV survivors do not report.

**Objective:**

We aimed to determine the proportion of survivors of SGBV in Mayuge District, Uganda, who report SGBV and the factors associated with reporting.

**Methods:**

Using a cross-sectional study design, we analyzed data of SGBV survivors in eight villages in Mayuge district collected in a baseline survey of a larger experimental study. Data were analysed using Modified Poisson Regression.

**Results:**

Of the 723 participants, 65% were female. Only 31.9% had reported the SGBV experienced. Reporting was 43% lower among survivors aged 45 years and older (p-value = 0.003), and 41% lower among survivors with higher than a primary school education (p-value = 0.005). Likewise, reporting was 37% lower among survivors who relied on financial support from their partners (p-value = 0.001). Female survivors were also 63% more likely to report (p-value = 0.001), while survivors who were separated/widowed were 185% more likely to report than those who were never married (p-value = 0.006).

**Conclusions:**

Reporting of SGBV by survivors in Mayuge was found to below.

## Background

Sexual and Gender-Based Violence (SGBV), especially against women, remains a major public health problem worldwide. The term SGBV encompasses different forms of violence including sexual violence; physical and emotional violence by an intimate partner; harmful traditional practices; and socio-economic violence^1^. Globally, about 1 in 3 women aged 15 years and older have ever experienced physical and/or sexual intimate partner violence during their lifetime^2^. In Uganda, 22% of women and 8% of men have ever experienced some form of sexual violence^3^.

This violence is more prevalent in the rural and poorer parts of the country like Mayuge district^3^. It is fuelled by society attitudes and practices that promote gender inequality and put women in a subordinate position in relation to men^1^. In all its forms, SGBV is a violation of human rights and undermines the health and dignity of its survivors. Among women, intimate partner violence has been shown to be associated with HIV, sexually transmitted infections^4^, depressive symptoms and suicide attempts^5^. These effects are often worse if survivors do not report or seek help.

Reporting allows survivors of SGBV to access the medical, psychosocial and legal services they need to minimize the impact of the violence on their health and also allows perpetrators to be held accountable. Moreover, formal reporting of SGBV, for example to medical personnel, legal officers or community leaders, allows accurate estimation of the prevalence of the violence. This enables proper resource allocation towards interventions to reduce SGBV and provide appropriate care to survivors. However, data from Demographic and Health Surveys of 24 countries worldwide showed that only 7% of all women who had experienced gender-based violence reported to a formal source^6^. In Uganda, among survivors of sexual and physical violence, only 33% of the female survivors and 30% of the males were reported to have sought help, while 51% of the women and 49% of the men neither sought help nor told anyone about the violence^3^.

The barriers to reporting of SGBV include shame, guilt and stigma associated with, especially, sexual violence; lack of access to medical care^7^; concerns about confidentiality and being believed^8^; and barriers specific to seeking help from police like the fear of reprisal resulting from reporting^9^. Similarly, poverty and the costs associated with reporting of SGBV such as transport for the complainant also sometimes hinder reporting and lead to settling cases out of court^10^. Gender inequality has also been cited as a barrier to reporting of SGBV, especially among female survivors. This is because it renders a lot of women submissive and economically dependent on their male partners who may at times be the perpetrators of the violence.^11^

However, most previous research on SGBV reporting has focused on sexual and intimate partner violence, overlooking emotional and socio-economic violence. Other studies have only focused on specific vulnerable groups like the women^6^, the disabled^12^, and refuge populations 13, leaving the reporting behaviour of some groups ununderstood. This study, therefore, aimed to determine the proportion of survivors of emotional, physical, socio-economic and sexual violence who report the violence experienced and to determine the factors associated with reporting. This information will guide the design of interventions to enhance reporting of SGBV experienced.

## Methods

### Study design

A cross-sectional study design was used. The study used baseline data collected in a larger experimental study testing a community intervention to reduce SGBV in Mayuge (14).

### Study setting

Data were collected in October 2019 from eight villages in Mayuge, a district in the Eastern part of Uganda. The 2014 National Population and Housing Census put the total population of the district at 473,239 people, with 51.6% of the total population being female and the majority (58.7%) aged between 0 to 17 years^15^.

### Participants

The study included men and women aged 18 years and older who reported experiencing at least one form of SGBV. Those aged 15 to 17 years were also included if they were considered emancipated minors who were already married, had children or were pregnant. Participants were only included if they normally resided in the visited households; were domestic servants who had slept in the households for at least five nights a week; or were visitors who had slept in the household for at least the past four weeks.

### Sampling procedure

For the larger experimental study, all villages in Mayuge were stratified as either rural or urban and a random sample of four villages was drawn from each stratum. Quota sampling was then used to select an equal number of men and women from each village, with one eligible person per household randomly selected for interview.^14^ To select participants for inclusion in this cross-sectional study, all those who reported at least one form of SGBV in the baseline survey of the experimental study were included in the analysis.

### Study size

A total of 995 participants were included in the larger experimental study^14^. Data of all 723 participants who reported experiencing at least one form of SGBV in their lifetime in the larger study were included in this study.

### Variables

Participants were considered to have experienced SGBV if they had experienced either physical violence by an intimate partner, emotional violence by an intimate partner, sexual violence or socio-economic violence in their lifetime. Physical, emotional and sexual violence were assessed using questions adopted from Garcia-Moreno, Jansen^16^. Socio-economic violence was used to refer to the denial of access to social and economic rights based on one's gender^1^. It was defined as: the respondent being denied access to education, health assistance or remunerated employment because of their gender; being denied property rights because of their gender^1^; being prohibited by their partner from getting a job, going to work, trading or earning money; or their partner taking their earnings against their will^17^.

The outcome of interest, reporting of SGBV was assessed using the question: “Have you told anyone about the violence you experienced?” Participants who responded “Yes” were further asked who they had told, and those who had reported to a health worker, police, social services organisation, local leader, religious leader or a counsellor were considered to have reported formally. Survivors who reported to friends, family members and neighbours were considered to have reported to informal sources.

Potential covariates identified from previous literature were also measured. These included; the participants' age, gender, education, marital status, residence, perceived availability of support from their family of birth, their main source of financial support and their attitudes towards wife-beating. The attitudes towards wife-beating were assessed using the question, “In your opinion, is a husband justified in hitting or beating his wife in the following situations: if she goes out without telling him; if she neglects the children; if she argues with him; if she refuses to have sex with him; or if she burns the food”. Respondents who answered “Yes” to at least one of the scenarios were coded as having attitudes accepting of SGBV.^3^

### Data collection

Data on SGBV, its reporting and the covariates were collected using an interviewer-administered electronic questionnaire through a household survey. This questionnaire was administered in Lusoga, the language predominantly spoken in the area. The interviews were conducted by male and female research assistants, depending on the respondents' preference.

### Statistical analysis

Data were analysed using Stata Version 14 (StataCorp LP, TX, USA). Modified Poisson Regression with robust standard errors was used for both bivariate and multivariable analysis, and associations were presented as prevalence ratios and adjusted prevalence ratios (APRs) with their 95% confidence intervals (CIs). Only variables with p-values less than 0.2 at bivariate analysis were considered for inclusion in the multivariable model. A p-value of less than 0.05 was considered statistically significant for all analyses.

### Ethics approval and consent to participate

Ethical approval for the study was sought from Makerere University School of Public Health Institutional Review Board (Protocol Number: 702) and the Uganda National Council for Science and Technology (Reference Number: HS457ES). Written informed consent was also sought from all study participants. Data were collected according to the World Health Organisation's Ethical and Safety Recommendations for Research on Domestic Violence Against Women^18^.

## Results

### Participant characteristics

Of the 723 participants who reported having experienced at least one form of SGBV in their lifetime, 65% were female (Table 1). Only 16% of the women and 30.4% of the men had attained education higher than primary school. The majority, 78.7% of the women and 80.6% of the men, were married or living with their partners. Most of the men (93.3%) derived their main source of financial support from their incomes, while 45.5% of women relied on their partners for financial support. Among the women, 48.1% found wife-beating justifiable in at least one situation as compared to 26.5% of the men. The commonest type of violence among both sexes was emotional while sexual violence was the least prevalent.

**Table 1:** Participant characteristics (N = 723)

	Women	Men
Characteristic	n (%)	n (%)
n (%)	470 (65.0)	253 (35.0)
Age		
15 to 24	136 (28.9)	59 (23.3)
25 to 34	166 (35.3)	79 (31.2)
35 to 44	80 (17.0)	56 (22.1)
45+	88 (18.7)	59 (23.3)
Highest education level attained		
None	168 (35.7)	54 (21.3)
Primary^a^	227 (48 .3)	122 (48.2)
Higher^b^	75 (16.0)	77 (30.4)
Marital status		
Never married	16 (3.4)	30 (11.9)
Married/living with partner	370 (78.7)	204 (80.6)
Separated/widowed	84 (17.9)	19 (7.5)
Residence		
Rural	208 (44.3)	141 (55.7)
Urban	262 (55.7)	112 (44.3)
Main source of financial support		
Personal income	184 (39.2)	236 (93.3)
From family and friends	72 (15.3)	11 (4.3)
From partner	214 (45.5)	6 (2.4)
Find wife-beating justifiable in at least one circumstance		
Yes	226 (48.1)	67 (26.5)
Life-time experience of SGBV by type of violence		
Emotional violence	400 (85.1)	217 (85.8)
Socioeconomic violence	247 (52.5)	99 (39.1)
Physical violence	218 (46.4)	59 (23.3)
Sexual violence	162 (34.5)	48 (19.0)

a Primary school is equivalent to about 7 years of formal education.

b Higher: Secondary school (6 years) or tertiary education.

### Reporting of SGBV

Of the 723 survivors of SGBV, only 231 (31.9%) had reported the violence they had experienced to anyone, and of these, 12.5% had reported formally.

### Factors associated with reporting of SGBV

At bivariate analysis, reporting of SGBV appeared to be more prevalent among females and among the separated/widowed, and less prevalent among survivors who had higher than a primary school education and those who did not find wife-beating justifiable in any situation. (Table 2)

**Table 2:** Bivariate and multivariable analysis of factors associated with reporting to any source. (N = 723)

	SGBV Reporting					
	No (n=492)	Yes (n=231)	Bivariate analysis		Multivariable analysis	
Variable	n (%)	n (%)	Crude PR (95% CI)	P-value	APR (95% CI)	P -value
**Age in years**						
15 to 24	137 (70.3)	58 (29.7)	1.00		1.00	
25 to 34	153 (62.4)	92 (37.6)	1.26 (0.96–1.65)	0.090	1.17 (0.89–1.53)	0.247
35 to 44	91 (66.9)	45 (33.1)	1.11 (0.81–1.54)	0.517	0.91 (0.66–1.25)	0.563
45+	111 (75.5)	36 (24.5)	0.82 (0.58–1.18)	0.286	0.57 (0.39–0.83)	**0.003**
Gender						
Male	198 (78.3)	55 (21.7)	1.00		1.00	
Female	294 (62.5)	176 (37.5)	1.72 (1.33–2.24)	<0.001	1.63 (1.22–2.18)	**0.001**
Highest education level attained						
None	134 (60.4)	88 (39.6)	1.00		1.00	
Primary	238 (68.2)	111 (31.8)	0.80 (0.64–1.00)	0.050	0.80 (0.64–1.00)	0.053
Higher	120 (78.9)	32 (21.1)	0.53 (0.38–0.75)	<0.001	0.59 (0.41–0.85)	**0.005**
Marital status						
Never married	39 (84.8)	7 (15.2)	1.00		1.00	
Married/living with partner	403 (70.2)	171 (29.8)	1.96 (0.98–3.92)	0.058	1.87 (0.90–3.88)	0.092
Separated/widowed	50 (48.5)	53 (51.46)	3.38 (1.67–6.86)	0.001	2.85 (1.35–5.99)	**0.006**
Residence						
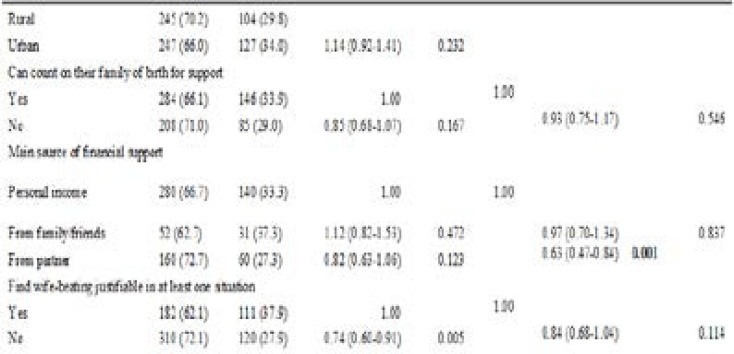

However, at multivariable analysis, the prevalence of reporting was found to be 43% lower among survivors who were aged 45 years and older as compared to those who were less than 25 years old, APR = 0.57 (95% CI: 0.39, 0.83; p-value = 0.003). Reporting was also 41% lower among those who had attained higher than primary school education, as compared to those with no education at all, APR = 0.59 (CI: 0.41, 0.85; p-value = 0.005). Likewise, reporting was 37% lower among survivors who relied on their partners for financial support as compared to those who relied on their incomes, APR = 0.63 (CI: 0.47, 0.84; p-value = 0.001).

On the other hand, reporting was 63% higher among females than males, APR=1.63 (CI: 1.22, 2.18; p-value = 0.001). Survivors who were separated from their partners or widowed were also more than twice as likely to report the violence experienced as compared to those who were never married, APR = 2.85 (CI: 1.35, 5.99; p-value = 0.006).

## Discussion

We found the level of reporting of SGBV among survivors in Mayuge to be low, even lower than the 39.9% reported for female survivors of gender-based violence in 24 developing countries, including Uganda. However, formal reporting in our study was higher than the 7% recorded for these survivors.6 Reporting was also found to be more prevalent among women. This could be related to the patriarchal nature of many societies in Uganda (19). These uphold masculinity idealisations that condition male survivors of SGBV to remain silent about the violence experienced 20. Similar underreporting of SGBV has been documented in conflict-afflicted Democratic Republic of Congo among male rape survivors because of feelings of shame, stigma and emasculation21. Such society ideals of what is expected of a man hinder reporting of SGBV.

We found reporting to be lower among survivors aged 45 years and older as compared to those younger than 25. This finding may imply that, because of awareness creation, survivors have gradually become more knowledgeable about the importance of reporting and the available reporting channels. And, because life-time experience of SGBV was used in the assessment, the younger survivors who had more recent experiences of SGBV may have been more likely to report than those 45 years and older who may have experienced violence over a longer period. However, this result is contrary to findings by Palermo, Bleck6 where increasing age was shown to be associated with an increased likelihood of reporting among female survivors of gender-based violence in developing countries.

Reporting was also lower among survivors who had higher than a primary school education as compared to those with no education at all. Older and educated community members are usually looked up to by others, and this lower reporting may be related to the need to uphold this social standing. However, education creates awareness about the available reporting channels and would be expected to increase reporting, although this is contrary to our findings. Palermo, Bleck^6^ also found contradicting results about the effect of education on reporting of SGBV: in some countries like Nigeria, women with no education were less likely to report SGBV while in others like Tanzania and Philippines, women with higher education were less likely to report. Reporting was lower among survivors who relied on their partners for financial support as compared to those who relied on their income. In Uganda, employed women and men were found to be more likely than the unemployed to seek help to end violence 3. Women's financial dependency on their partners was also cited as a barrier to help-seeking by abused women in Rwanda because their partners controlled the family resources and made decisions about how money could be spent^11^. In our study, more women reported relying on their partners for financial support than men, further highlighting the need for economic empowerment of those most vulnerable to SGBV to increase their autonomy in relationships and ability to report and seek help in case of violence.

Survivors who were separated from their partners or widowed had a higher prevalence of SGBV reporting than those who were never married. This is comparable to what Palermo, Bleck^6^ found in 15 of 24 developing countries, where formerly married women were more likely to report the violence experienced than currently married women. The 2016 UDHS also found separated and divorced men and women to be most likely to seek help for the violence experienced, as compared to the married and those who were never-married^3^. This could be related to the ignorance among the married about the fact that marital rape is a crime that needs to be reported^11^. Umubyeyi, Persson^11^ also reported that family matters, including violence and abuse against women, in Rwanda were considered secrets to be retained within the family, which also affected reporting and help-seeking among survivors of intimate partner violence. As such, divorced and separated survivors may feel less bound by such expectations of secrecy and may be more open to speaking out about the violence experienced both in and outside marriage. We, however, did not observe any statistical difference in reporting between the married and those who were never married.

## Limitations

Since a quantitative cross-sectional study design was used, we were unable to establish temporality between the exposure variables and reporting. We were therefore limited in our understanding of the true relationship between variables like marital status and the reporting of SGBV. Furthermore, we were limited in our ability to understand the institutional barriers to reporting. More research using qualitative methods is recommended to further explore and understand the barriers to SGBV reporting.

Additionally, because SGBV is a sensitive issue, underreporting of the violence experienced by the study participants could have occurred. We, however, minimised this by training research assistants to conduct interviews in private spaces, away from any interruptions to encourage accurate reporting.

## Conclusion

The reporting of SGBV was found to be low among survivors in Mayuge. Reporting was more prevalent among female survivors and those who were separated from their partners or widowed. On the other hand, it was less prevalent among survivors aged 45 years and older, those with higher than a primary education and among those who received their main source of financial support from their partners.

We recommend interventions that promote dialogues about SGBV and its reporting, especially among the men, older survivors and the educated to encourage reporting and promote help-seeking to stop the violence and to increase utilisation of the available support services for survivors. We also recommend economic empowerment for those most vulnerable to SGBV to increase their autonomy in relationships and ability to report the violence experienced.

Additionally, leaders at the community level, such as the religious and local leaders, should be empowered with information and resources to effectively provide support to survivors of SGBV in their communities, as these are sometimes the survivors' first points of contact.
